# Ethical Dilemmas in End-of-Life Care: A Psychological and Anesthetic Perspective

**DOI:** 10.7759/cureus.111202

**Published:** 2026-06-20

**Authors:** Joshua Nmezi

**Affiliations:** 1 Anaesthesia / Clinical Psychology, Imo State University, Owerri, NGA; 2 Anaesthesia, Pilgrim Hospital, Boston, GBR

**Keywords:** advance directives, anaesthesia, clinical psychology, cultural competence, double effect, end-of-life care, ethics, moral distress, palliative care

## Abstract

End-of-life care presents some of the most complex and emotionally challenging ethical dilemmas in modern medicine. For anesthetists and clinical psychologists, these dilemmas intersect at the critical juncture where physiological support meets psychological suffering. This editorial advances the novel argument that the psychological distress inherent to the dying process constitutes a specific clinical entity (existential suffering), which requires interdisciplinary management distinct from standard symptom control. It explores the major ethical challenges encountered in end-of-life care, including decisions about withholding and withdrawing life-sustaining treatment, do-not-resuscitate orders, medically administered nutrition and hydration, palliative sedation, and requests for assisted dying. The specific, practical roles of the anesthetist and clinical psychologist are detailed within a collaborative model, moving beyond generalities of "symptom management" and "grief support." Principles of biomedical ethics (autonomy, beneficence, non-maleficence, and justice) provide a framework for analyzing these dilemmas. Culturally specific decision-making frameworks for Nigerian and African contexts, including family-centric consensus models and communication scripts, are introduced. Practical recommendations are offered in the form of specific communication tools and assessment prompts for clinicians navigating these challenging situations and self-care to prevent moral distress and burnout.

## Editorial

End-of-life care represents one of the most profound and challenging areas of clinical practice. It is a domain where technical medical expertise intersects with deep psychological, spiritual, and existential questions about the meaning of life, the nature of suffering, and the boundaries of medical intervention [1]. For clinicians working at the bedside of dying patients, the decisions made in these final hours, weeks, and months carry immense weight, affecting not only the quality of a patient's death but also the long-term psychological well-being of families and the clinicians themselves [2].

This editorial takes the position that the psychological distress experienced by many dying patients is not merely a secondary symptom of physical pain but a primary form of suffering (existential suffering) that demands its own therapeutic framework. Unlike anxiety or depression, which are often managed pharmacologically, existential suffering arises from threats to identity, meaning, connection, and autonomy in the face of non-being [3]. It is a position that challenges the purely biomedical model of end-of-life care, arguing that anesthetists and clinical psychologists must work as equal partners to address this distinct form of distress.

Anesthetists and clinical psychologists occupy unique positions in end-of-life care. The anesthetist's role extends beyond pharmacology and terminal weaning to include proactive communication about the trajectory of physiological decline. They are uniquely positioned to translate complex physiological data (e.g., patterns of tachypnoea, changes in oxygenation) into plain language for families, thereby alleviating the terror of the unknown. Specific actions include performing a targeted "symptom autopsy" to distinguish terminal agitation from existential distress, leading family meetings to explain the process of terminal extubation in real-time and reframing the cessation of monitoring not as withdrawal of care but as a transfer from aggressive treatment to comfort-focused presence [4].

The clinical psychologist's role is equally specific. Beyond processing grief, psychologists can administer brief, validated screening tools for existential distress, such as the Demoralization Scale, during routine palliative reviews. They facilitate "dignity therapy" protocols-brief, evidence-based interventions that help patients create a legacy document for their families [5]. In the final hours, they can guide families through "rituals of connection," such as reading favourite passages, playing specific music, or facilitating life review, thereby transforming the dying process from a purely medical event into a humanized, relational one.

The four principles of biomedical ethics articulated by Beauchamp and Childress (autonomy, beneficence, non-maleficence, and justice) provide a foundational framework for analysing ethical dilemmas in this context [6]. Respect for autonomy requires honouring patients' rights to make informed decisions about their own medical care, including refusal of treatment even when that refusal may lead to death. Beneficence compels clinicians to act in patients' best interests, interpreted at the end of life as providing comfort, relieving suffering, and preserving dignity when curative interventions are no longer appropriate [6]. The obligation to non-maleficence, to first do no harm, becomes complex when interventions intended to be beneficial also carry risks--a situation addressed by the doctrine of double effect, which distinguishes intended from merely foreseen consequences [7]. Justice requires equitable distribution of healthcare benefits and burdens, raising questions about resource allocation and access to palliative care services [6].

Withholding and withdrawing life-sustaining treatment remains one of the most fundamental ethical questions in end-of-life care. The consensus in medical ethics holds that there is no moral distinction between withholding and withdrawing treatment--if a treatment is not benefiting a patient or its burdens outweigh its benefits, there is no ethical obligation to continue it regardless of when it was initiated [8]. Practical decisions should be based on the patient's expressed wishes, likely prognosis, burdens of treatment relative to benefits, and input from family members. When patients lack capacity, surrogate decision-makers must make decisions based on what the patient would have wanted [9].

Do-not-resuscitate orders are designed to prevent inappropriate application of cardiopulmonary resuscitation in situations where it would be futile or contrary to a patient's wishes [10]. Discussions should occur early in the clinical course, clarifying that a do-not-resuscitate order refers only to resuscitation and does not imply withdrawal of other treatments. The rationale must be explained in terms of avoiding harm rather than framed as giving up, as patients and families may misunderstand these orders to represent withdrawal of all care.

The question of medically administered nutrition and hydration at the end of life is particularly emotionally charged, as food and water carry deep symbolic meaning [11]. These are medical interventions subject to the same ethical considerations as any other treatment and may be withheld or withdrawn if they are not providing benefit. The symbolic meaning must be acknowledged in discussions, and families may require extensive support when navigating these decisions. Regardless of the decision about artificial nutrition, mouth care, and small amounts of oral intake for comfort should always be provided [11].

Palliative sedation involves the use of sedative medications to relieve intolerable suffering when other treatments have failed, intended to reduce consciousness to relieve suffering, not to hasten death [12]. It is ethically distinct from euthanasia, as its intention is symptom relief. The doctrine of double effect provides the framework: an action with both good and bad effects is ethically permissible if the action itself is good, the good effect is intended, the bad effect is not the means to the good effect, and the good effect outweighs the bad effect [7]. Palliative sedation should be reserved for extreme, refractory symptoms, with decisions involving the whole team and, where possible, the patient and family [12].

Requests for assisted dying remain among the most controversial areas of end-of-life care, though increasingly legal in specific jurisdictions [13]. This editorial takes the position that the professional response should not be reflexive opposition or referral but a structured exploration. When requests arise, clinicians should listen empathetically, explore the reasons behind the request (often reflecting fear of pain, loss of dignity, or becoming a burden), ensure all palliative options have been explored, document discussions carefully, and seek support from colleagues and ethics committees [13].

The COVID-19 pandemic fundamentally altered end-of-life care, introducing novel ethical challenges. The requirement for infection control led to unprecedented restrictions on family presence, creating a new syndrome of "lonely dying," characterized by heightened existential distress, delirium, and a sense of abandonment [14]. Anesthetists, as managers of airways and ventilators, were thrust into the role of primary emotional witnesses for patients who died without family. Post-pandemic, the ethical obligation has shifted to embedding "virtual presence" (tablets for video calls, recorded messages from family) as a standard component of end-of-life protocols and to providing targeted debriefing for staff who experienced repeated traumatic, isolated deaths [15].

The role of clinical psychologists in end-of-life care has also evolved. Beyond traditional grief therapy, psychologists now employ brief, evidence-based interventions. "Dignity therapy," developed by Chochinov, involves a trained therapist guiding a patient through a series of questions to create a formal legacy document for their family--a process proven to reduce demoralization and increase a sense of purpose in the final weeks of life [5]. "Meaning-centered psychotherapy," adapted from the work of Viktor Frankl, helps terminally ill patients maintain a sense of meaning in the face of death [16]. These are not generic "support" but active, structured psychological treatments that can be delivered in as few as three to four sessions.

For anesthetists, the specific technical and communication skill of "terminal weaning" from mechanical ventilation requires formal training and ethical rehearsal. This involves not only the pharmacological titration of sedatives and paralytics but also the co-ordination of a family-centred ritual: the extubation itself should be paused, explained, and framed as a compassionate act, not a failure. Post-extubation, the anesthetist remains present to manage signs of air hunger with opioids, translating the patient's physiological responses into a narrative of comfort for the family [4].

The integration of anesthetic and psychological expertise is best understood through a tiered collaborative model. Tier 1 (Basic) involves regular interdisciplinary team meetings where the anesthetist reports on physiological trends and the psychologist reports on patient coping and family dynamics. Tier 2 (Integrated) involves joint family meetings where both clinicians are present: the anesthetist explains the "what" (e.g., "The breathing machine is doing the work, and we are going to let the body follow its natural course"), and the psychologist addresses the "why" and "how" (e.g., "You can stay by the bedside, talk to her, and she will hear your voice even if she cannot respond"). Tier 3 (Specialist) involves collaborative research into interventions that target the biological-psychological interface of suffering, such as the use of low-dose ketamine for both pain and existential distress.

Cultural beliefs profoundly shape attitudes toward death and dying. In Nigerian and African contexts, many cultures emphasize collective decision-making over individual autonomy, with family elders expected to make decisions [17]. This is not a barrier to be overcome but a distinct ethical framework (sometimes termed "family-centric consensus"), which requires the clinician to identify the legitimate decision-maker (e.g., the oldest son, the family matriarch) and conduct separate "pre-meetings" with family factions before the formal discussion. The goal is not to extract a patient's individual preference in isolation but to build a collective consensus that the family will support after the patient's death, thereby preventing complicated bereavement.

In some cultures, telling a patient they are dying may be seen as causing harm. This requires clinicians to balance respect for cultural norms with obligations of truth-telling. A pragmatic middle ground is the "request-based disclosure" model: the clinician asks the family, "Does the patient want to know the full details of their illness?" and "Who should tell them, and how much?" [18]. This respects the family's role while leaving the door open for the patient to request information.

Spirituality and religion play central roles, with traditional beliefs, Christianity, and Islam influencing attitudes toward suffering and death [18]. In the Nigerian context, specific spiritual concerns include the need for rituals of blessing or anointing, the role of prayer in symptom management, and fears about a "bad death" that could affect the patient's spiritual trajectory. A simple cultural assessment tool for the clinician includes three questions: "Are there rituals or spiritual leaders you would like present?"; "Are there specific fears about what happens after death?"; and "Do your beliefs forbid or encourage any medical treatments (e.g., blood transfusions, organ donation, sedation)?" [19].

Resource limitations in low- and middle-income countries create specific ethical dilemmas not seen in high-income settings. A shortage of opioids (due to both cost and restrictive prescribing laws) can force clinicians to choose between leaving a patient in pain or using non-standard, less effective agents. The scarcity of trained psycho-oncologists and palliative care specialists means that the responsibility for psychosocial support falls onto overburdened nurses and junior doctors. In this context, the ethical principle of justice demands that clinicians advocate for systemic change: lobbying for essential palliative care medicines on the WHO Essential Medicines List, training non-specialists in basic psychological first aid, and adapting Western psychotherapy protocols for delivery by lay health workers [20].

To move from generic recommendations to actionable practice, the following specific tools are proposed. For breaking bad news: a sample script: "I am worried that your body is no longer responding to the treatments that help most people. I believe we are now in a different phase-one where our goal is not to cure but to make sure you are not suffering and that you are not alone. Is that something we can talk about?" For cultural assessment: a prompt: "In your community, how are decisions about serious illness usually made? Is it the patient alone, the family together, or a specific person?" For managing requests for hastened death: a response: "I hear how much you are suffering. My job is to help you. I cannot do what you are asking but let me tell you what I can do to control your pain, to keep you from feeling abandoned, and to make sure your life has meaning until its natural end."

For individual clinicians, developing self-awareness about personal values and responses to death is essential, along with seeking education in palliative care and ethics. A specific starting point is a personal "ethics audit": reviewing three recent patient deaths and asking, "What did I do well?" "What do I regret?" and "What will I do differently next time?" For clinical teams, establishing clear processes for decision-making, holding regular multidisciplinary meetings, and providing debriefing after difficult deaths fosters collaboration and support [21]. The "Schwartz Rounds" model (a structured, multidisciplinary forum where clinicians discuss the emotional and social aspects of patient care, not the clinical details) has been shown to reduce moral distress and increase team cohesion [22]. For communication, involving patients and families early, using clear language, and documenting discussions thoroughly ensures understanding and continuity. For organizations, providing education, establishing ethics committees, ensuring access to palliative care, and recognizing moral distress as an occupational hazard are fundamental responsibilities [23].

Ethical dilemmas in end-of-life care are inevitable, complex, and deeply human. They arise at the intersection of technical medical expertise and profound psychological, spiritual, and existential concerns. This editorial has argued that these dilemmas require more than the application of generic principles; they demand a specific, interdisciplinary clinical response that targets existential suffering as a distinct entity. Anesthetists must move beyond pharmacology to become skilled communicators of physiological decline, and clinical psychologists must move beyond grief therapy to deliver active, structured interventions like dignity therapy. For anesthetists and clinical psychologists, these dilemmas demand not only clinical skill but also ethical reasoning, emotional resilience, and the capacity for compassionate communication. The post-COVID era has not only added new layers of complexity, from the trauma of lonely dying to the resource constraints of low-income settings, but it has also clarified the non-negotiable need for human presence at the end of life. Ultimately, navigating these dilemmas requires multidisciplinary collaboration, clear communication, and a commitment to supporting not only patients and families but also the clinicians who accompany them through the final chapter of life. By integrating the skills of anesthesia and clinical psychology, clinicians can offer comprehensive care that addresses both physical suffering and psychological distress, honoring the dignity of each person who dies in their care.
